# Influence of Manatees' Diving on Their Risk of Collision with Watercraft

**DOI:** 10.1371/journal.pone.0151450

**Published:** 2016-04-06

**Authors:** Holly H. Edwards, Julien Martin, Charles J. Deutsch, Robert G. Muller, Stacie M. Koslovsky, Alexander J. Smith, Margaret E. Barlas

**Affiliations:** 1 Fish and Wildlife Research Institute, Florida Fish and Wildlife Conservation Commission, 100 Eighth Ave. SE, St. Petersburg, FL, 33710, United States of America; 2 Southeast Ecological Science Center, U.S. Geological Survey, 7920 NW 71st Street, Gainesville, FL, 32653, United States of America; 3 Wildlife Research Laboratory, Fish and Wildlife Research Institute, Florida Fish and Wildlife Conservation Commission, 1105 SW Williston Road, Gainesville, FL, 32601, United States of America; Sonoma State University, UNITED STATES

## Abstract

Watercraft pose a threat to endangered Florida manatees (*Trichechus manatus latirostris*). Mortality from watercraft collisions has adversely impacted the manatee population’s growth rate, therefore reducing this threat is an important management goal. To assess factors that contribute to the risk of watercraft strikes to manatees, we studied the diving behavior of nine manatees carrying GPS tags and time–depth recorders in Tampa Bay, Florida, during winters 2002–2006. We applied a Bayesian formulation of generalized linear mixed models to depth data to model the probability (*P*_*t*_) that manatees would be no deeper than 1.25 m from the water’s surface as a function of behavioral and habitat covariates. Manatees above this threshold were considered to be within striking depth of a watercraft. Seventy-eight percent of depth records (individual range 62–86%) were within striking depth (mean = 1.09 m, max = 16.20 m), illustrating how vulnerable manatees are to strikes. In some circumstances manatees made consecutive dives to the bottom while traveling, even in areas >14 m, possibly to conserve energy. This is the first documentation of potential cost-efficient diving behavior in manatees. Manatees were at higher risk of being within striking depth in shallow water (<0.91 m), over seagrass, at night, and while stationary or moving slowly; they were less likely to be within striking depth when ≤50 m from a charted waterway. In shallow water the probability of a manatee being within striking depth was 0.96 (CI = 0.93–0.98) and decreased as water depth increased. The probability was greater over seagrass (*P*_*t*_ *=* 0.96, CI = 0.93–0.98) than over other substrates (*P*_*t*_ = 0.73, CI = 0.58–0.84). Quantitative approaches to assessing risk can improve the effectiveness of manatee conservation measures by helping identify areas for protection.

## Introduction

Watercraft collisions have posed a significant threat to the endangered Florida manatee (*Trichechus manatus latirostris*) in recent decades [1, 2]. Marine mammal populations including endangered North Atlantic right whales (*Eubalaena glacialis*), humpback whales (*Megaptera novaeangliae)*, Atlantic bottlenose dolphins (*Tursiops truncates)* and others worldwide, are being harmed by vessel strikes [3, 4]. Relative to other threats (e.g., loss of warm-water habitat, crushing in water-control structures, entanglement, and red tide), watercraft strikes exert the greatest adverse impact on manatee population growth rate and the risk of quasi-extinction [1]. Reducing mortality due to collisions with watercraft may be necessary for the continued recovery of this endangered marine mammal.

In 2007, the U.S. State of Florida’s Manatee Management Plan [5] identified reducing human-caused manatee mortality, including those from watercraft strikes, as an important objective for managing the population in perpetuity throughout Florida. Efforts to reduce the number of manatees being struck by boats have been implemented since 1979, when the first manatee protection zone was designated. Although these protective measures have likely helped reduce mortality from collisions with watercraft [6], the threat to the subspecies still exists [6, 7].

The distribution and movements of manatees within their home range, including vertical movements, are influenced by activities like traveling, resting, thermoregulating, mating, calving, foraging, and drinking. Risk to manatees increases when they spend more time near the water’s surface. It may also increase when they are unable to move or dive away from an approaching watercraft, or when in areas were manatees and boats are most likely to co-occur. Boats and manatees are known to use all parts of Tampa Bay (or Florida waters in general including both fresh and saltwater areas). Given the average draft category of motorized watercraft commonly using some of Florida’s waterways (Tampa Bay, 0.28–1.09 m; [8]), a manatee within that depth from the surface that directly encounters a moving watercraft (i.e., is passed over by it) is likely to be struck, injured, and perhaps killed.

To quantify the problem of watercraft strikes on manatees, we used manatee dive profiles to 1.) estimate the probability of a manatee being within striking depth of a watercraft (≤1.25 m); and 2.) assess the influence of manatee behavior and habitat features on this probability.

Time-depth dive profiles of marine mammals have almost exclusively been used to describe foraging behavior or to assess bioenergetics [9, 10]. A few studies have used dive data to estimate the probability of detecting animals during surveys [11, 12, 13, 14] and to examine how surface foraging of North Atlantic right whales increases the risk of collision with vessels [15]. Diving behavior has been used to assess manatee behavioral response to approaching boats [16]. However, detailed diving behavior of manatees as it relates to habitat features and manatee behavior has never been described. We used manatee depth data, analyzed with generalized linear mixed models (binomial mixed-effects models; GLMM) within a Bayesian framework to estimate the probability of a manatee being within 1.25 m of the water’s surface (henceforth referred to as striking depth) in relation to a suite of environmental and behavioral covariates. In addition, we used GPS data to summarize manatees’ behavior around a power station’s (manatee winter aggregation site) no-boat-entry discharge canal.

## Materials and Methods

### Study area

**Fig 1:**
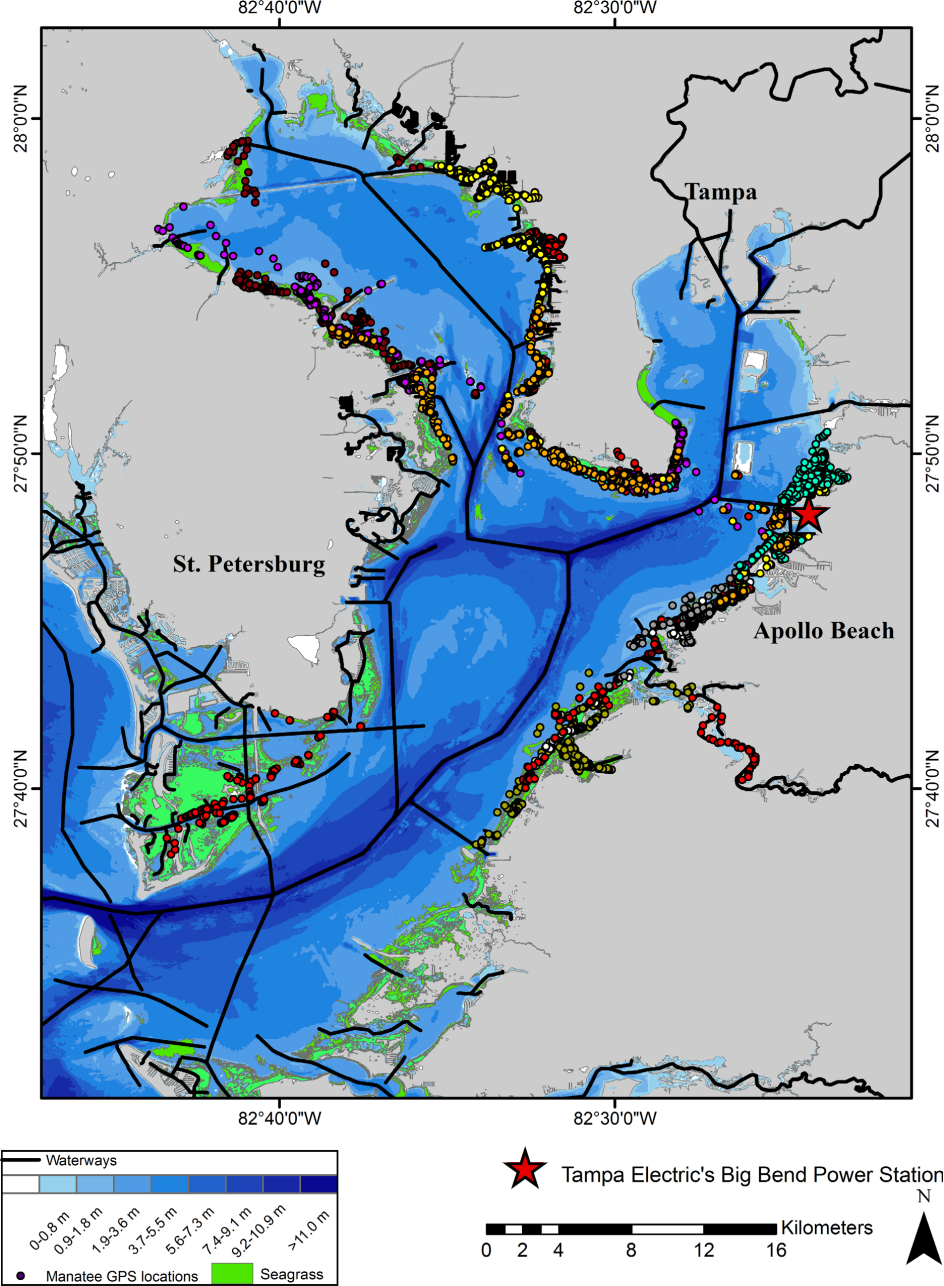
Map of Tampa Bay, FL, U.S.A. with bathymetry, known waterways, seagrass and GPS locations (by color) for nine manatees.

Our study was conducted during four winters (December–March) in Tampa Bay, a large shallow estuary in west-central Florida, U.S.A. (~1,030 km^2^; Fig 1). Tampa Bay contains 78 km of dredged channels, and it averages ~4 m deep with a maximum depth of 27 m, occurring at its mouth. The bay typically has two unequal high and low tides (mixed, semidiurnal tides) each day with an average tidal range of 0.7 m [17].

Manatees are poorly adapted to cold [18, 19] and depend on natural and industrial sources of warm water for survival in winter [20]. There are three industrial warm-water sources in Tampa Bay; the principal site used by manatees is the Tampa Electric’s Big Bend Power Station near Apollo Beach (Fig 1). Aerial surveys recently documented >600 manatees using this plant’s warm-water discharge in winter (FWC unpublished data). The discharge canal is a no-entry area for boats, though the warm-water plume used by manatees for thermoregulation extends several hundred meters beyond the no-entry zone, into waters used by boaters.

### Field methods

In every December from 2002–2005, manatees were arbitrarily captured in or near the Big Bend Power Station’s thermal discharge canal as part of concurrent studies on manatee detectability, movements, and attendance patterns at an industrial warm-water aggregation site in winter [21, 12, 22]. Permission to conduct this work in Tampa Bay and in the Tampa Electric’s Big Bend Power Station was granted by the United States Fish and Wildlife Service (permit# MA773494), State of Florida’s Bureau of Protected Species Management, and the Tampa Electric Company. Time–depth–temperature data loggers (model LTD-100 Archival Data Logger, Lotek Inc., St. Johns, Newfoundland, Canada) and Argos-linked GPS satellite telemetry tags (Telonics Inc. models TMT-240 and TMT-460, Mesa, Arizona) were attached to 16 animals, of which 9 provided adequate depth data for use in this study. The remaining seven TDR recorders were either lost or damaged, or returned data of insufficient quality for this project. The telemetry tags acquired GPS positions at 20-min (winter 2002–03) or 15-min (winters 2003–04, 2004–05, 2005–06) intervals throughout the 24-hr cycle. Success rate for the GPS fixes ranged from 71.0% to 85.2%. A rooftop test of accuracy of the GPS locations from one TMT-240 tag and one TMT-460 tag found the median error to be 5.0 m and 7.8 m, respectively; 98% and 95% of locations were within 25.0 m of the true location, respectively [21]. The TDR recorded pressure (converted to depth; accuracy of 0.1 m) and temperature every 30 sec (winter 2002–03) or 5 sec (winters 2003–04 to 2005–06). The TDRs were attached 0.3 m from the base of a 1.5-m nylon tether used to secure the telemetry tag to the peduncle belt (around the base of the tail); this placement approximated the manatee’s back when the animal was horizontal. A common orientation of a manatee suspended in water is one in which the head and back are slightly higher than the peduncle. The TDR was placed on the tether near the point at which the body meets the tail (below the back and head). The average body pitch angle from 18 tagged manatees [16] was used to estimate the vertical distance from the peduncle to the manatee’s back (0.16 m), which is usually the part of the manatee closest to the surface. Based on this calculation, we added 0.16 m to the maximum for average-draft watercraft (1.09 m; see above) to calculate a conservative striking depth threshold (1.25 m).

### Data analyses

#### Manatee behavior around a warm-water site

We used manatee GPS data and the full dive profile data set from TDRs (not reduced for autocorrelation as in the GLMM analyses; see below) to summarize the behavior of manatees when they were outside the power plant’s discharge canal from December through March. Locations of manatees in the canal were excluded from our analyses because watercraft are prohibited there. We determined the duration of sorties, (i.e., travel bout to and from the power plant discharge canal), maximum distance traveled from the plant, movement speed, mean manatee depth, and proportion of dive records within striking depth. We also quantified the proportion of depth records over seagrass (a staple of the manatee diet), in water-depth bins, and by time of day.

#### Probability of being within striking depth

Our goal was to estimate the probability of a manatee being above a specific water depth threshold (*P*_*t*_; within striking depth vs. deeper than striking depth) in relation to behavior and environmental features (categories used as co-variates). We applied GLMMs [23] implemented with a Bayesian approach (using non-informative priors) to estimate *P*_*t*_ (posterior mean) as a function of covariates using a logit link. The number of TDR records ≤ the threshold was assumed to follow a binomial distribution with the size parameter for the distribution set at the total number of records. Our covariates included water depth category, presence of seagrass, travel speed category, time of day, and proximity to a known waterway. Individual manatees were treated as a random effect in the model. Due to collinearity among covariates, a separate GLMM analysis was conducted for each covariate. The Bayesian analyses were run using program WinBUGS v 1.4 [24] and the R (version 3.0.3, [25]) package R2WinBUGS [26]. Our analyses were similar to those used by Hagihara et al. [13] in their study of the dugong (*Dugong dugon*), but they used a maximum-likelihood approach (R package lme4, [27]) to analyze diving data. Kéry [28] suggests that the estimates of variance may be more accurate (return less biased estimates of uncertainty) with the Bayesian implementation.

#### Time–depth and covariate data

Covariates considered likely to influence manatee diving behavior were selected based on manatee ecology and habitat requirements. Presence of seagrass, water depth bin, and proximity to a waterway were determined using ArcGIS (Esri, Redlands, California) and telemetry locations. Speed category was assigned by examining the time interval between successive GPS fixes. If two or more successive GPS fix attempts were unsuccessful, if GPS locations were >30 minutes apart, and if the track in GIS showed that the manatee’s location changed enough to indicate quick, directed movement, then the associated TDR records were considered fast travel. Locations <30 minutes apart that were stationary, or indicated milling about, or slow directed movement were considered slow travel. Winter daytime hours were defined as 0700–1759 hr EST and nighttime hours as 1800–0659 hr EST. To be considered near a waterway, two or more consecutive GPS points had to be ≤50 m from the nearest known waterway (charted navigational routes and unmarked pathways digitized from NOAA nautical charts; Fig 1). Water depth was coded using three categories (Cat 1, ~0.00–0.91 m; Cat 2, ~0.92–1.82 m; Cat 3, ~1.83–3.66 m) referenced to the NAVD88 vertical datum (0.163 m below local mean sea level; [29]). There were too few records for depths >3.66 m to be used as a category in the analyses. Water depth was not adjusted for tidal fluctuations; therefore, manatee depths may be deeper or shallower than the range indicated for that category. Because of small scale variation in bathymetry (e.g., deep holes in shallow water) a manatee could dive deeper than the maximum depth of a bin. The higher of the two daily high tides during the months of this study generally occurred between 1600–0500 hr EST; water depth was also influenced by wind speed and direction. TDR records that could not be attributed to a depth category because the track from the preceding to the next GPS location crossed a depth contour line were unassigned.

The binary coding of the covariates for each depth record was done manually by examining each GPS location in a GIS to determine the approximate location of the manatee at the time of the TDR reading. For example, if two sequential GPS locations were located over seagrass, a 1 was assigned to the covariate field that corresponded to the TDR data within that time period. Sorties of <30 min were excluded from the data set. Because this manual process was very labor intensive on these very large data sets, three-fourths of the remaining sorties were randomly sampled for inclusion in our GLMM analyses.

Dive data are obviously autocorrelated; to eliminate the effects of autocorrelation, we selected points at 10-minute intervals [11] for the GLMM analyses. Although combinations of the covariates seem to have relevant interactions, many are correlated (e.g., water depth and seagrass); those interactions were not included in our analyses. In some cases, interactions were not evaluated because of data limitations (e.g., small number of records in deep water). The interaction between time of day and travel speed was the only interaction considered in the GLMM. To quantify the difference between estimates (e.g., *P*_*t*_ over seagrass versus *P*_*t*_ not over seagrass), we estimated the effect size (ES) as the arithmetic difference between estimates and presented the 95% credible interval (CI). A 95% CI[ES] that did not overlap zero was considered statistically significant [21].

## Results

### Summary of manatee movement behavior

The amount of time that the nine tagged manatee spent outside of the no-boat-entry discharge canal of the Big Bend Power Station averaged 49% (Table 1). The maximum time between sorties was 2.5–6.1 days (Table 1). Individual means of the maximum distances traveled for each sortie from the power plant were relatively short (3.6–11.0 km), and mean movement velocity was about a third of the mean maximum velocity of 0.90 m/sec.

**Table 1:** Summary of manatee age–sex class, TDR deployment duration, and movement and diving behavior. The number, duration and distance of sorties, maximum time between sorties, mean and maximum velocities outside of the discharge canal and standard deviations are given for the entire GPS tag deployment duration. Time spent outside of the power plant’s no-boat-entry canal, mean and maximum dive depths, and proportion of depth records ≤1.25 m, are given for the period of TDR data collection.

Manatee ID (age class-sex)	TDR deployment (days)	Time outside power plant (hrs [%])	Max. time between sorties (days)	Mean sortie length (hrs)	Mean max. distance (km [SD])	Mean velocity (m/sec [SD])	Max. Velocity (m/sec)	Mean Depth (m [SD])	Max. Depth (m)	Proportion of Records <1.25 m
TTB105(SubA-F)	18.5	217.0(49)	6.1	14.1(38.8)	4.7(8.5)	0.35(0.16)	0.76	1.44(1.25)	15.32	0.62
TTB108(Ad-F)	39.8	429.7(45)	3.4	39.5(35.9)	10.8(12.1)	0.36(0.17)	1.05	0.84(0.68)	14.92	0.86
TTB101(Ad-M)	68.0	874.8(54)	5.0	17.3(24.3)	6.3(7.0)	0.27(0.17)	0.83	1.11(0.97)	14.04	0.78
TTB122(Ad-F)	17.2	221.7(54)	5.1	14.7(7.2)	6.4(3.4)	0.42(0.16)	0.92	1.02(0.57)	6.39	0.85
TTB119(SubA-F)	41.8	369.0(37)	3.4	15.5(32.1)	3.6(6.5)	0.29(0.13)	0.83	1.02(0.89)	16.02	0.76
TTB115(Ad-F w/calf)	41.8	645.0(64)	2.5	29.3(81.1)	6.7(9.7)	0.31(0.13)	0.91	0.98(0.47)	6.43	0.86
TTB094(SubA-F)	70.1	931.7(55)	5.8	24.6(25.2)	11.0(7.7)	0.49(0.16)	1.10	1.09(0.66)	6.04	0.77
TTB099(Ad-M)	53.5	417.3(33)	3.5	13.2(9.5)	4.1(3.1)	0.27(0.17)	0.77	1.08(0.66)	7.99	0.76
TTB093(SubA-F)	75.1	887.4(49)	4.5	17.8(19.8)	8.5(7.6)	0.40(0.17)	0.93	1.22(1.24)	16.16	0.75
Mean	47.3	554.8(49)	4.4	20.7	6.9	0.35	0.90	1.09	11.48	0.78

(Ad = adult, SubA = subadult, M = male, F = female)

### Summary of dive records

**Fig 2:**
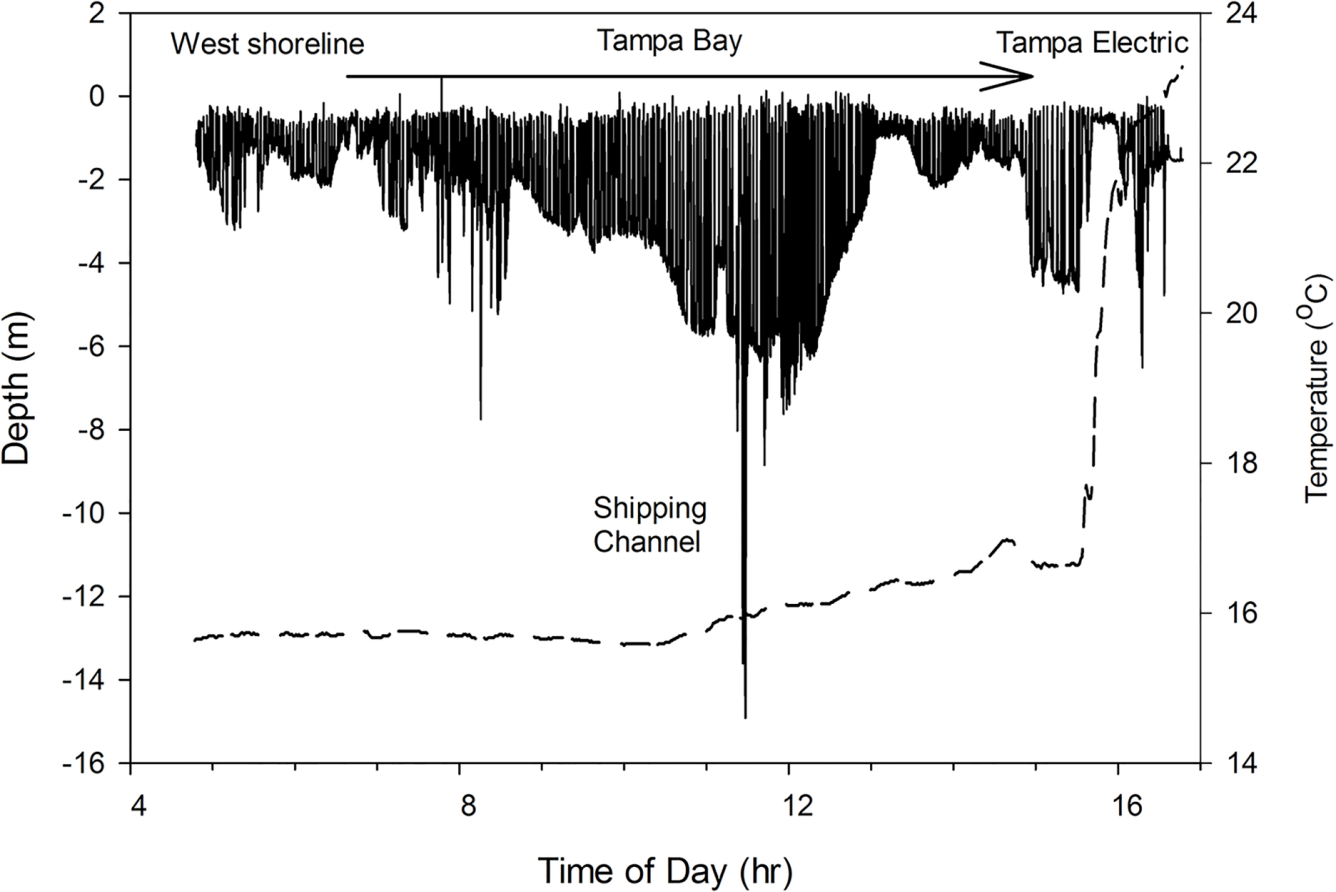
Dive profile of manatee TTB108 crossing Tampa Bay (west shoreline to Tampa Electric Company’s Big Bend Power Station) from 04:47:55 to 16:47:15 EST, January 31, 2004. Dashed line depicts water temperature recorded by time-depth recorder.

**Fig 3:**
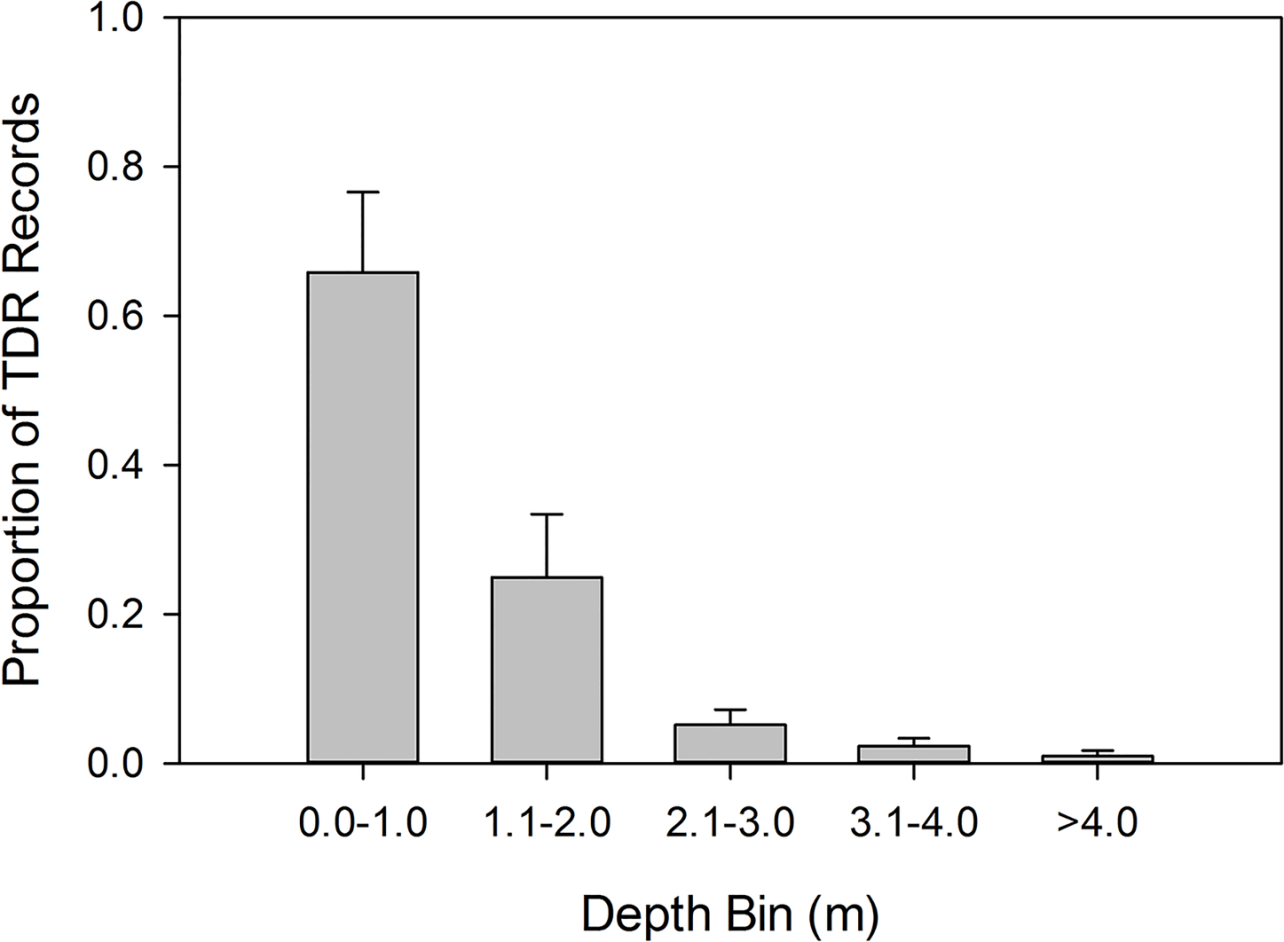
Proportion of depth records (n = 1,557,957) collected by time-depth recorder on nine manatees in depth bins, showing distribution of manatee depths.

**Fig 4:**
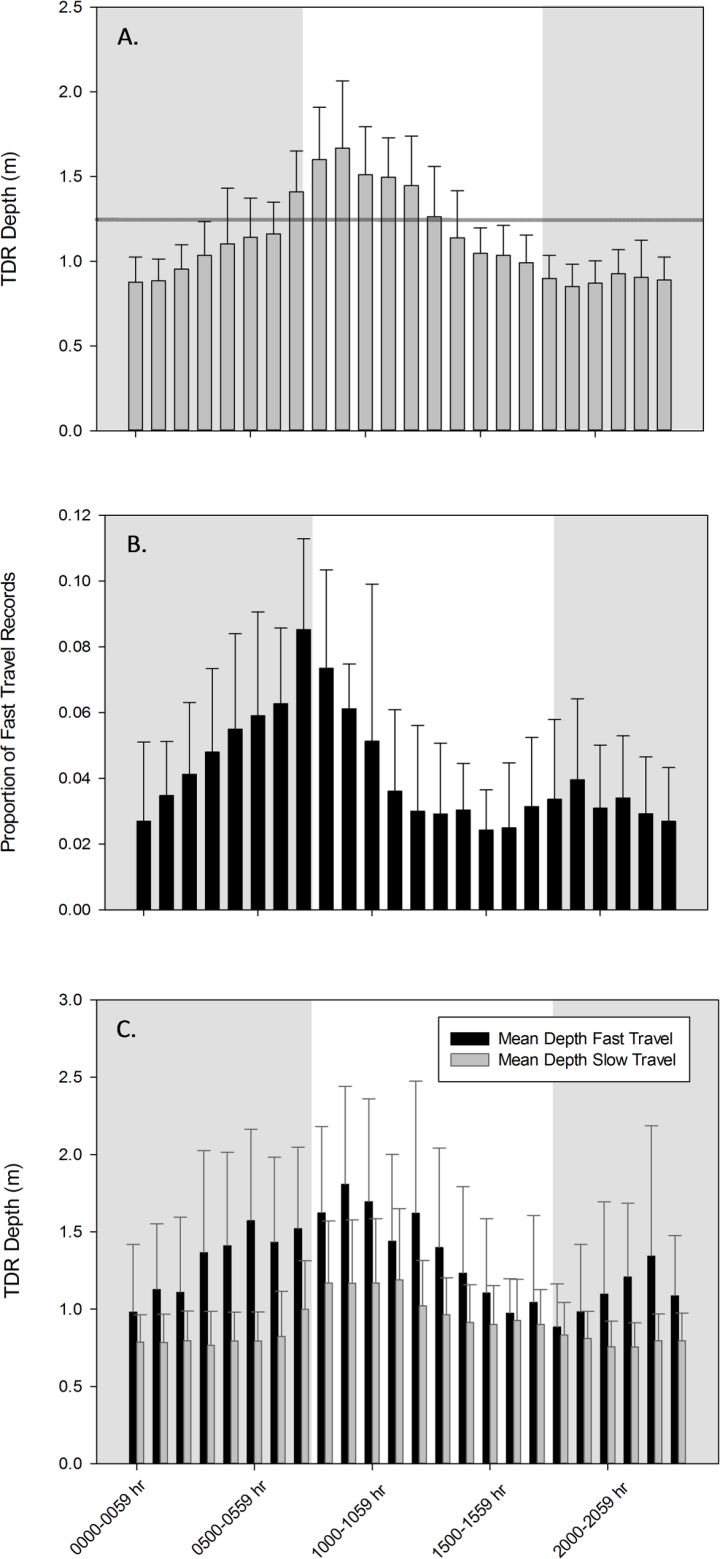
A.) Mean depth (m) for all dive records recorded by time-depth recorders (n = 9) by hour with standard deviations; horizontal line is striking depth. B.) Proportion of fast travel movement records by hour with standard deviations. C.) Mean depth (m) during fast and slow travel movements with standard deviations.

Individual TDRs operated for 17–75 days (Table 1). Mean depth was 1.09 m (SD = 0.17), with maximum depth ranging from 6.0–16.2 m across individuals. The greatest depths recorded for 5 manatees (14.0–16.2 m) corresponded to depths recorded only in shipping channels or other dredged channels (Fig 1). A sample dive profile of a manatee crossing such a shipping channel illustrates that under some circumstances manatees make consecutive dives to the bottom even in the deepest areas of the bay (Fig 2). The depth distribution of TDR records indicates that manatees spent a large proportion of time close to the surface, where they were at risk of being struck by watercraft (Figs 3 and 4A). The overall proportion of depth records within our striking depth ranged from 0.62 to 0.86 among individuals (mean = 0.78; Table 1).

**Fig 5:**
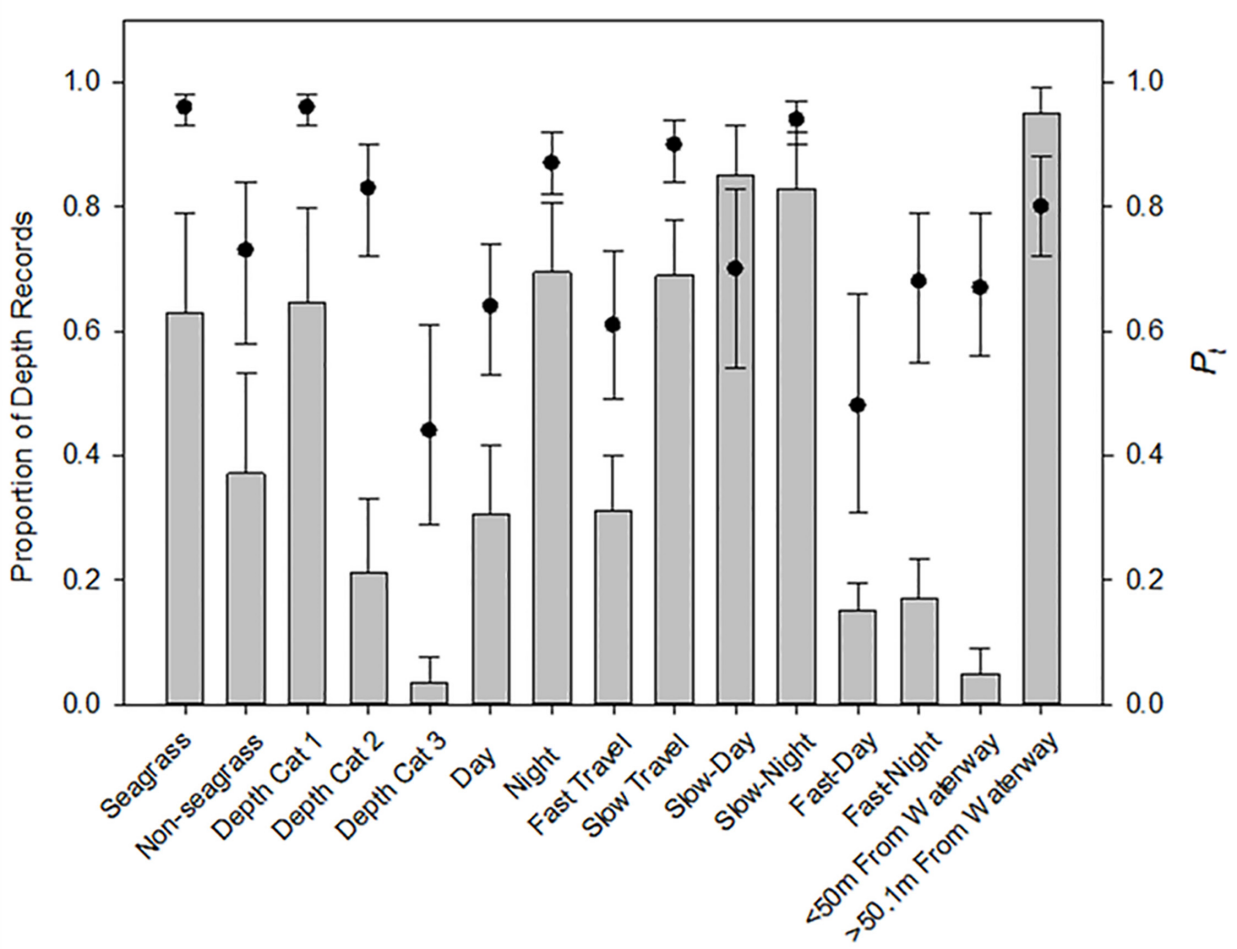
Proportion of depth records (vertical bars), with standard deviations, in relation to substrate, movement category, time of day, depth categories, and proximity to a waterway. GLMM probabilities (*P*_*t*_) of being within striking depth (≤1.25m) are shown for each with 95% credible intervals (circles).

Shaded bars in Fig 5 show the proportion of depth records for each covariate. A covariate could not be determined for 25% of seagrass records and 37% of water depth records. Fast travel occurred at night in 17% of the records and during the day in 15% of the records; 56% of fast travel records were in Depth Cat 1, 36% were in Cat 2, and 7% were in Cat 3. For slow travel 72% were in Cat 1, 26% were in Cat 2, and 2% were in Cat 3.

Manatee depth showed a diel pattern, with hourly mean TDR depths being greatest during the morning hours (~1.4–1.6 m) and lowest at night (~0.8–1.1 m) (Fig 4A). This diel variation in hourly mean depths was most pronounced during fast travel movements, as shown in Fig 4C.

### Probability of being in striking depth

The probability of a manatee being within striking depth was high in Depth Cat 1 (*P*_*t*_ = 0.96, CI = 0.93–0.98) but declined with increasing water depth (Fig 5; Table 2). Presence in a seagrass bed had a similar effect on risk (*P*_*t*_ = 0.96, CI = 0.93–0.98), which was significantly greater than when a manatee was over bottom types other than seagrass (*P*_*t*_ = 0.73, CI = 0.58–0.84; ES = 0.23, ES(CI) = 0.14–0.35). Manatees spent significantly less time within striking depth during fast travel (*P*_*t*_ = 0.61, CI = 0.49–0.73) than during slow travel (*P*_*t*_ = 0.90, CI = 0.84–0.94; ES = 0.29, ES(CI) = 0.21–0.36). A graph of all TDR depths by hour supports that finding (Fig 4C). Fast travel occurred most often in early morning hours (Fig 4B), which correspond to the times when manatees are likely returning to the power plant waters. Time of day had a significant effect on the proportion of time spent within striking depth, which was significantly higher at night (*P*_*t*_ = 0.87, CI = 0.82–0.92) than during the day (*P*_*t*_ = 0.64, CI = 0.53–0.74; ES = 0.23, ES(CI) = 0.18–0.29). Although the estimate of *P*_*t*_ was greater for fast travel at night than during the day, the CI overlapped (Table 2). The estimate of *P*_*t*_ was significantly less for locations ≤50 m from a known waterway (*P*_*t*_ = 0.68, CI = 0.56–0.79) than when farther away (*P*_*t*_ = 0.81, CI = 0.72–0.88, Fig 5); although the CI for *P*_*t*_ overlapped, the CI of ES did not overlap with zero (Table 2).

**Table 2:** Results of generalized linear mixed models (GLMMs) showing the probability (*P*_*t*_) of a manatee being above a 1.25-m threshold for striking depth by watercraft and effect size (ES) with standard deviations (SD) and 95% credible intervals (CI) in relation to habitat features, time of day, and travel speed category.

Model	Fixed Effect		*P*_*t*_	SD	CI	ES	ES(SD)	ES(CI)
1	Seagrass	Seagrass	0.96	0.01	0.93–0.98			
		Nonseagrass	0.73	0.07	0.58–0.84	0.23	0.05	0.14–0.35
2	Water depth	Cat 1(0.00–0.91m)	0.96	0.01	0.93–0.98			
		Cat 2(0.92–1.82m)	0.83	0.05	0.72–0.90	0.13	0.03	0.08–0.21
		Cat 3(1.83–3.66m)	0.44	0.08	0.29–0.61	0.38	0.05	0.28–0.46
3	Time of day	Day	0.64	0.05	0.53–0.74			
		Night	0.87	0.03	0.82–0.92	0.23	0.03	0.18–0.29
4	Travel speed	Slow	0.90	0.03	0.84–0.94			
		Fast	0.61	0.06	0.49–0.73	0.29	0.04	0.21–0.36
5	Travel speed, day	Slow day	0.70	0.08	0.54–0.83			
		Fast day	0.48	0.09	0.31–0.66	0.21	0.02	0.16–0.25
6	Travel speed, night	Slow night	0.94	0.02	0.90–0.97			
		Fast night	0.68	0.06	0.55–0.79	0.26	0.04	0.18–0.35
7	Distance to waterway	≤50 m from waterway	0.68	0.06	0.56–0.79			
		>50.1 m from waterway	0.81	0.04	0.72–0.88	0.13	0.02	0.09–0.18

## Discussion

Tagged manatees in Tampa Bay, FL U.S.A. during winter spent an average of 78% (range 62–86%) of their time, when outside of the power plant’s refuge, near the surface of the water (≤1.25 m), where they were at the greatest risk of being struck by a watercraft. This statistic alone emphasizes the vulnerability of Florida manatees to collisions in the shallow-water habitats they typically occupy.

### Relationship between habitat features and proportion of time manatees spent near the surface

Tagged manatees spent most of their time in very shallow water (Depth Cat 1) (Fig 5). As expected, the probability of a manatee being at striking depth when in the shallowest depth bin was very high, at 0.96 (Table 2). Manatees would have little opportunity to escape being struck by a watercraft passing overhead in this depth category because it is usually shallower (≤0.9 m) than striking depth. The probability of a manatee being within striking depth decreased with increasing water depth (Table 2). Water depth was undetermined for 37% of the TDR records, often due to lack of a GPS location. Many of those records were probably from the deeper categories because GPS fix attempts were more likely to be unsuccessful when a manatee was either diving or traveling quickly across open water and pulling the tag’s antenna underwater. When outside of the power plant refuge, 63% of TDR records were over seagrass beds, where there was a 0.96 (SD = 0.01) probability that a manatee would be above our threshold for striking depth. The probability of a manatee being in striking depth over bottom types other than seagrass (both shallow and deep waters) was less (0.73), but the effect of seagrass presence on risk is confounded with water depth, because seagrass in Tampa Bay is found in shallow waters (<2 m).

### Relationship between time of day, manatee activity, and proportion of time manatees spent near the surface

The timing of a manatee movements to and from the power plant waters, the water depths that it encountered, and its diving behavior had a temporal influence on the probability of it being within striking depth of a watercraft. The probability of a manatee being within striking depth was significantly greater at night than during the day (Table 2; Fig 5). Mean depth for all TDR records by hour supports this finding (Fig 4A). The difference in depth by time of day may be explained by both habitat features (e.g., water depth) and manatee behavior. Since many sorties away from the power plant are likely foraging bouts, traveling to and from feeding areas may influence a manatee’s risk of being struck by a watercraft. In winter, the daily higher high tides in Tampa Bay occur from 1600–0500 hr EST. Access to inshore seagrass beds during higher tides may explain why manatees generally leave the warm water of the power plant in the afternoon and evening and occupy shallow water at night [20, 30]. Time spent in shallow seagrass areas likely accounts for the higher probability of being within striking depth during the night than during the day. Daytime resting behavior may also influence dive depth, since resting often occurs on the bottom in deep areas, although surface resting is also common [12].

The probability that a manatee would be within striking depth during fast travel (*P*_*t*_ = 0.61, CI = 0.49–0.73) was significantly less than that during slow travel (*P*_t_ = 0.90, CI = 0.84–0.94; Fig 5). That is, manatees were at greater depths during fast travel movements (Fig 4C). The difference in mean depth by speed category varied by time of day, with the greatest differences in the early morning (see below; Fig 4C). Slow travel occurred more often in shallower water; 72% of slow travel records versus 56% of fast travel records were in Depth Category 1, where *P*_*t*_ was estimated to be 0.96.

During fast travel, the probability that a manatee would be within striking depth during the night was 0.68 (CI = 0.55–0.79); during the day it was 0.48 (CI = 0.31–0.66), one of the lowest estimates for any of the covariates in these analyses (Table 2), albeit still high from a risk perspective. The *P*_*t*_ credible intervals overlapped but the CI(ES) did not overlap zero. Mean depths during fast travel were greatest (close to about 1.5 m) from 0300–1359 hr (Fig 4C), which encompasses both nighttime and daytime periods; this likely influenced the GLMM results. When fast travel by time of day was considered, periods of fast travel occurred substantially more often in the morning (Fig 4B). This indicates that when a manatee engages in a morning trip, likely returning to the power plant, in many cases it travels faster and deeper, possibly due to its thermoregulatory need to return to warm water quickly after spending time in cooler water away from the plant at night. The difference in the proportion of time spent on the surface by time of day may reflect the manatee behavior of swimming more deeply or using deeper water areas, especially during morning trips back to the power plant’s warm water.

In cold weather, manatees must make quick movements to and from warm water and preferred feeding locations. Given the speed and distances manatees traveled (Table 1), a trip to or from foraging grounds takes several hours each direction. Swimming is energetically expensive, and marine mammals are known to modify their method of locomotion to reduce energetic costs [31, 32]. For a manatee that has been fasting inside the power plant discharge canal (in our case up to 6.1 days; Table 1) and traveling in cold water, or for one that will be fasting upon return to the power plant, minimizing the amount of energy expended getting to and from foraging grounds is important. Because drag is greater at the surface of the water than below it, staying submerged and using energy-efficient behaviors may reduce the overall cost of the long-distance movement [31, 32]. The dive profile of a manatee crossing one of the shipping channels in Tampa Bay while returning to the power plant (Fig 2), illustrates that under some circumstances manatees make consecutive dives to the bottom, even in the deepest areas of the bay, to possibly conserve energy. This is the first documentation of potential cost-efficient diving behavior in manatees. Sheppard et al. [33] noted a similar behavior in dugongs, which travel through the water column rather than near the surface and make repeated deep dives along their route. Energy-conserving behavior, and the deeper dives that result, could help explain the reduced amount of time manatees are within striking depth when they are traveling quickly, especially at times when they are returning to the power plant. This behavior may not occur as often in warmer months or when manatees are more stationary and quick movements to warm water are not so advantageous. Data collected on fluking behavior using a digital acoustic recording tag (DTAG) would be amenable for testing this hypothesis [16].

### Relationship between proximity to known waterways and proportion of time manatees spent near surface

Areas where boats travel, such as channels and other dredged waterways, present increased risk to manatees. Although we found that the probability of a manatee being within striking depth when ≤50 m of a waterway was less than when farther away (Fig 5), this result should be interpreted with caution because the CI of *P*_t_ overlapped and the ES(CI) was only marginally greater than zero (Table 2). In addition, all waterways may not have been depicted on the GIS map layer used for this analysis. The observed differences could be related to differences in water depth or behavior of manatees when in or near a waterway. Channels and dredged canals are deeper than surrounding waters. In this data set, manatee depths averaged about 0.3 m deeper when they were ≤50 m of a waterway (mean = 1.3 m, SD = 0.5). Manatees may spend more time deeper when in waterways as a means of minimizing risk of being hit by a boat. Another explanation is that deeper water serves as a travel corridor, and energetics favor deeper diving in those areas. The probability of a manatee encountering a boat in a dredged waterway when traveling could, however, increase the absolute risk to manatees and negate the advantage of spending more time at greater depths.

From the perspective of collision risk, we have provided the first estimate of the probability of manatees being within striking depth of a boat; an important quantity to derive measures of risk of collisions between boats and manatees [34, 35]. In isolation, this quantity cannot be used to identify areas with the highest risk of collision; nevertheless, it can contribute to a better understanding of the risk of collision process. Future studies can use our estimates to develop maps of risk of collision that consider other important parameters such as the probability of encounters between boats and manatees, and the probability of death given strike speed [35]. In this context, understanding how manatee diving behavior influences risk from collisions with watercraft and how it varies with habitat can help us identify areas that are most dangerous to manatees. These areas may be considered by managers for protection or regulation to benefit manatees, while possibly minimizing the impact of regulation on the boating community by targeting those areas with the greatest potential risk.
